# Handbook of Chemistry, Theoretical, Practical and Technical

**Published:** 1854-07

**Authors:** 


For sale by MILLER, ORTON & MULLIGAN. H.

---

ART. X.—*Handbook of Chemistry, Theoretical, Practical and Technical.* By F. A. ABEL, Prof. of Chem., etc., etc., and C. L. BLOXAM, formerly first assistant to the Royal College of Chemistry. With a preface by Dr. Huffman, and numerous illustrations on wood. Philadelphia: Blanchard and Lea. 1854.

One of the peculiarities of this republication of a recent English work, is that it has no American editor, further than the services of some unknown gentleman, who has given it his superintendence in the securing of typographical accuracy; a very important matter in a work of this character.

The title describes the book. It is not a text book proper; but is rather a compendium for use in the laboratory. It gives instruction in chemical manipulation, with only such a general account of chemistry as is involved in the operations of the laboratory, and in quantitative and qualitative analysis. The reactions of metallic oxides, of inorganic and organic acids, are arranged in groups, and precede the chapters on analysis.

Without any great familiarity with chemical literature, we conceive that this book fills a blank, by collecting in convenient form the various reactions and tests of chemical substances, unburdened by the usual lessons on elementary chemistry. For the teacher, the advanced student, or the practical chemist, it must be very valuable; while the practitioner of medicine will have not unfrequent occasion to refer to its pages.

